# Development and evaluation of a surgical skills lab for trainee surgeons: a 10-year experience at the Münster University Hospital

**DOI:** 10.1186/s12909-025-07064-3

**Published:** 2025-04-04

**Authors:** Annika Mohr, Jens Peter Hölzen, Sandra Stöppeler, Hans-Ullrich Spiegel, Daniel Palmes, Ralf Bahde, Linus Kebschull, Mazen A. Juratli, Benjamin Strücker, Andreas Pascher, Felix Becker

**Affiliations:** Department of General, Visceral and Transplant Surgery, University Hospital Münster, Waldeyerstr 1, Münster, 48149 Germany

**Keywords:** Surgical training, Postgraduate education, Surgical skills, Simulation training

## Abstract

**Background:**

Surgical training persists of intensive work, incessant practice, and, most importantly, experience. Owing to the changing surgical environment, increasing specialization, and rapid development of minimally invasive techniques, new innovative approaches in surgical training are necessary to achieve excellent postgraduate education. Here, we introduce a surgical skills lab that offers a multi-course program featuring a concise, modular curriculum comprising well-defined and simple-to-follow procedures, progressively moving surgical techniques from ex vivo to in vivo settings. The evaluation of the course was conducted by analyzing the participants’ self-assessment before and after the course.

**Methods:**

Over the time of ten years, we conducted one-day surgical training courses covering basic surgical techniques, gastrointestinal anastomosis, visceral resection techniques, and techniques in vascular surgery with a total of 348 participants. To assess differences in the self-evaluation of surgical skills before and after each course, a questionnaire (non-validated self-report 5-point Likert scale) was administered to each participant. Results were analyzed with t-test for paired samples.

**Results:**

Before the course, most participants had no practical knowledge of most exercises, and major help was needed. However, after training, the majority of participants were able to perform the surgical techniques independently with little or no assistance. Moreover, a statistical analysis comparing pre- and post-course self-assessment scores for surgical skills revealed significant improvements (*p* < 0.05) after the course.

**Conclusion:**

During the one-day course, it was possible to teach and perform diverse surgical procedures under the guidance of experienced surgeons. The independent reproducibility of the learned material after the course is not yet known, therefore, further investigation is necessary to provide additional information to improve the program. However, with this step-by-step training, we were able to conduct a successful teaching program, shown by the fact that the participants showed significant improvement. Thus, the training presented in this study can serve as a guide for teaching surgical skills outside of the operating room.

**Supplementary Information:**

The online version contains supplementary material available at 10.1186/s12909-025-07064-3.

## Background

Surgical training persists of intensive work, incessant practice, and, most importantly, experience. Therefore, to perform safe and effective surgery, a surgeon must complete several hundreds of hours of training to achieve expertise and appropriate skills in a wide range of operations [1–3]. The traditional training followed the established apprenticeship model in which trainees learn by observing and assisting experienced surgeons in the operating room [4–6]. However, over the past decades, there has been an increasing emphasis on reducing medical errors and ensuring patient safety within the surgical community, resulting in the reduction of the traditional model of “see one, do one, teach one” by Prof. Dr. William Halsted and trial and error [2, 7, 8]. Furthermore, the field of surgery is changing towards increasing specialization [7, 9]. Therefore, it is crucial to develop innovative strategies that focus on improving the training of surgeons and ensuring that they receive excellent postgraduate education, while providing safe patient care.

It has been shown that repetitive training can improve speed, fluidity as well as self-assurance in general surgical skills [1, 10, 11]. Moreover, superior technical skills are associated with lower complication rates for surgical procedures, thus improving patient outcome [12]. Currently, a wide range of simulation and training models exist that offer opportunities to teach and practice outside the operating room. The main goal of simulation training is to acquire and improve the skills required to perform surgical procedures without compromising patient safety [1, 5]. However, more complex surgical procedures necessitate an in vivo environment to safely learn the required surgical skills [13].

To date, most surgical departments have yet to establish a program for systematically teaching open surgery outside the operating room especially for assistant doctors, as the process of setting up the learning module is lengthy, the necessary equipment can be costly, and training itself takes a considerable amount of time. Moreover, simply repeating a task without receiving feedback from an experienced surgeon does not aid in retaining or enhancing skills.

In the Department of General, Visceral and Transplant Surgery, University Hospital Muenster, protocols and educational concepts for teaching specific surgical techniques under the supervision of experienced senior surgeons have been in place for more than a decade for in-house training. A group of practicing senior surgeons developed a step-by-step curriculum covering basic surgical techniques, gastrointestinal anastomosis, visceral resection techniques, and techniques in vascular surgery focusing on individual learning and deliberate practice to teach surgical proficiency. The overall idea was to develop a practical course (Basic Surgical Skills) with a clear and easy-to-understand curriculum, in which specific surgical techniques were first taught theoretically, then trained ex vivo and finally transferred to complex surgical procedures under in vivo conditions in animals. Based on our expertise, we propose the development and evaluation of a comprehensive training program for junior medical professionals, including interns, residents and medical students composed of four structured modules. This study aims to evaluate the impact of the training program on the participants by considering a self-assessment given to the participants before and after the course.

## Material and methods

### Study design

This study was conducted as a retrospective cohort analysis of prospectively collected questionnaire-based data generated during the ten years (2009–2018) of the hands-on Basic Surgical Skills courses conducted at the Department of General, Visceral and Transplant Surgery, University Hospital Muenster. The course's structure and objectives as well as the teaching materials and equipment utilized remained consistent over the 10-year study period. Each course of surgical training lasted for one day (8 h). Participants could choose between the following four modules:

1. Basic surgical techniques
2. Visceral resection techniques
3. Gastrointestinal anastomosis
4. Techniques in vascular surgery

The course was non-mandatory and offered to junior medical professionals, including interns and residents. Each course could be taken independently of each other. To assess the differences in self-evaluation of surgical skills before and after each course, a questionnaire (Supplementary Fig. 1) was created for this study using a 5-point scoring system (1 = no theoretical or practical knowledge, 2 = no practical but theoretical knowledge, 3 = performance with major help, 4 = independent performance with little help, and 5 = independent performance without help). Although the limitations of using non-validated self-report Likert scales are acknowledged, the selection was based on several scientific considerations including simplicity, reliability, and compatibility. Anonymous and non-mandatory questionnaires were distributed to the participants before and after the course. All participants were informed about analysis of data.

### Teaching program

The teaching program was developed and supervised by experienced senior surgeons. To learn the specific surgical techniques proposed in the curriculum, each course began with a theoretical lecture. For incorporation into a logical working algorithm and for understanding practical implementation, it is necessary to have a strong theoretical background [14, 15]. Following the theoretical lectures, each training module was designed to include a variety of practical tasks, beginning with synthetic tissues and cadaver organs in the skills lab and progressing to animal surgery with living animals.

### Basic surgical techniques

**Fig. 1:**
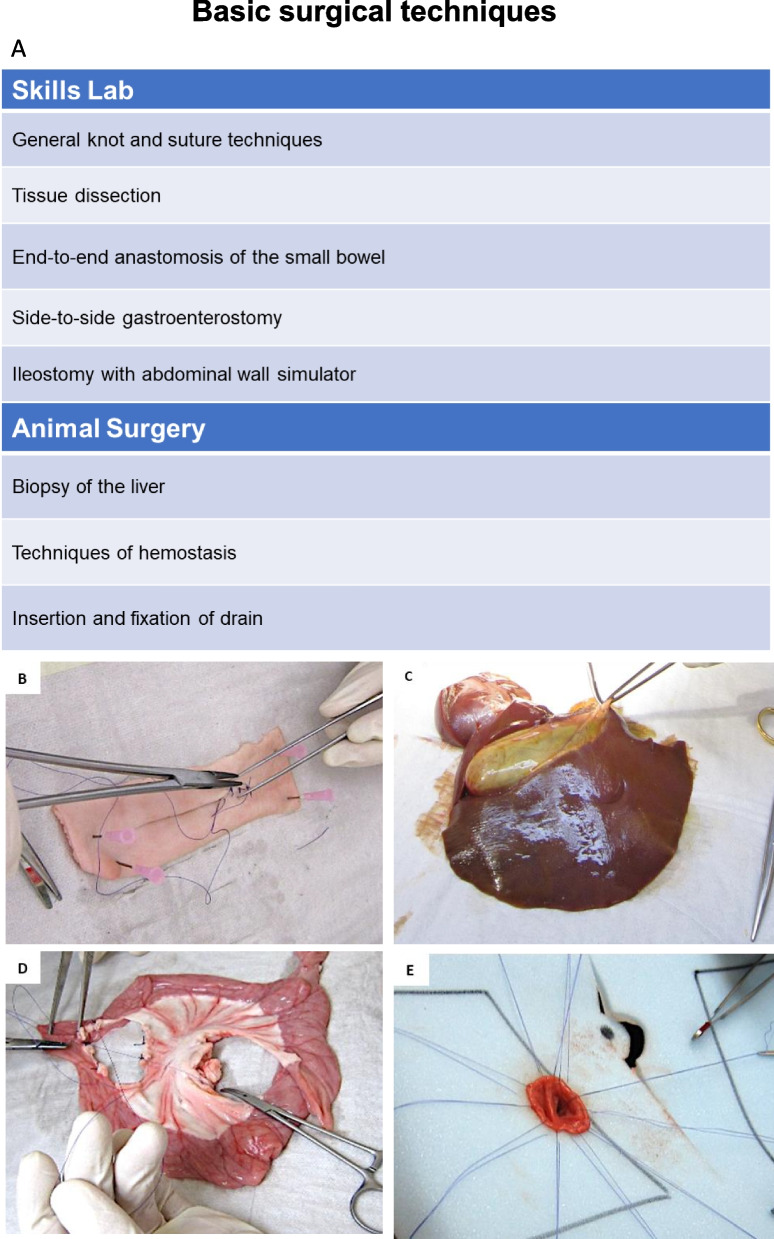
Basic surgical techniques. **A** Table of exercises in the skills lab ex vivo and animal surgery in vivo **B** Exemplary picture of the exercise “General knot and suture techniques” **C** Exemplary picture of the exercise “Tissue dissection” **D** Exemplary picture of the exercise “End-to-end anastomosis of the small bowel” **C** Exemplary picture of the exercise “Ileostomy with abdominal wall simulator”

The educational goal in this module was to learn basic surgical techniques starting ex vivo in the skills lab with general knot and suture techniques using knot boards and pig skin (Fig. 1B) following tissue dissection using pig livers (Fig. 1C). For the practice of gastrointestinal anastomosis the participants used porcine small bowel and stomach specimen to train end-to-end anastomosis of the small bowel (Fig. 1D) and side-to-side gastroenterostomy. The last exercise in the skills lab was a laparotomy and ileostomy training using an abdominal wall simulator and bowel from the pig (Fig. 1E). In the next step, training was transferred to the animal surgery performing in vivo biopsies of the liver, techniques of hemostasis and insertion and fixation of drains.

### Visceral resection techniques

**Fig. 2:**
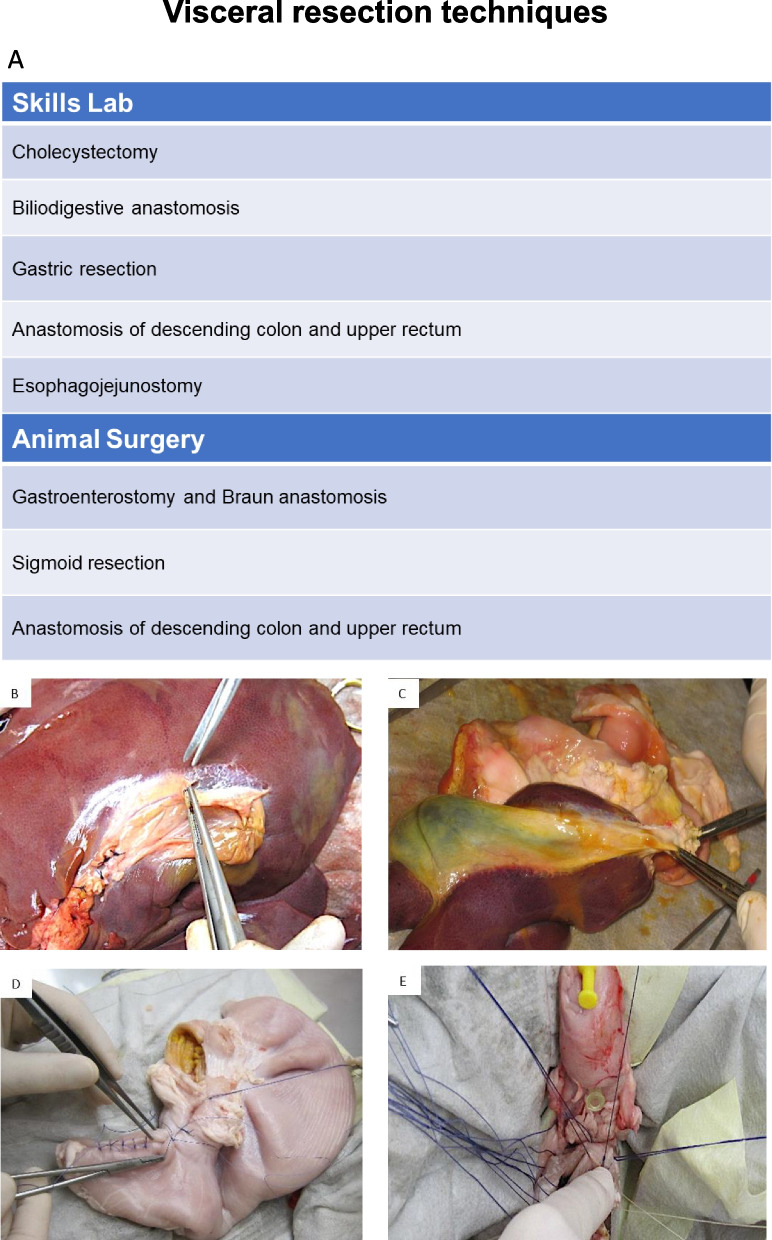
Visceral resection techniques. **A** Table of exercises in the skills lab ex vivo and animal surgery in vivo. **B** Exemplary picture of the exercise “Cholecystectomy” **C** Exemplary picture of the exercise “Biliodigestive anastomosis” **D** Exemplary picture of the exercise “Gastric resection” **E** Exemplary picture of the exercise “Esophagojejunostomy”

Similar to “ Basic surgical techniques”, the training of “ Visceral resection techniques” started with the ex vivo exercises in the skills lab with porcine organs (Fig. 2). First, the participants performed a cholecystectomy (Fig. 2B) followed by a biliodigestive anastomosis (Fig. 2C). The next exercise was a gastric resection (Fig. 2D). After that, anastomosis of the descending colon and the upper rectum was trained with a circular stapler device. The last exercise in the skills lab was a hand sewn esophagojejunostomy (Fig. 2E) after which exercises were continued in vivo. The first in vivo exercise was a gastroenterostomy and Braun anastomosis followed by a sigmoid resection. Lastly, an anastomosis of the descending colon and the upper rectum was trained with a circular stapler.

### Gastrointestinal anastomosis

**Fig. 3:**
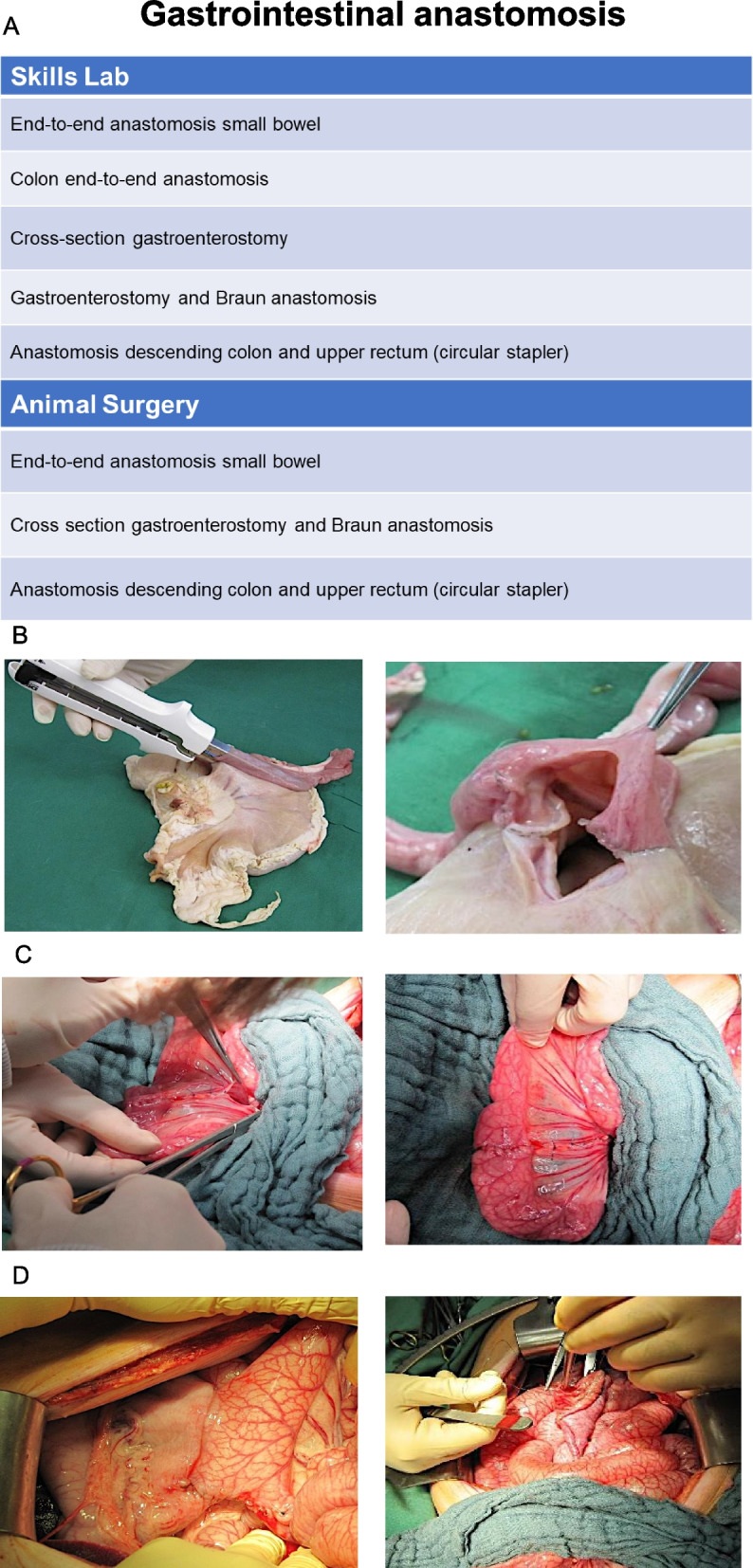
Gastrointestinal anastomosis. **A** Table of exercises in the skills lab ex vivo and animal surgery in vivo. **B** Exemplary picture of the exercise “Cross section gastroenterostomy (Skills Lab) **C** Exemplary picture of the exercise “end-to-end anastomosis small bowel” **D** Exemplary picture of the exercise “Cross section gastroenterostomy (in vivo)”

The module gastrointestinal anastomosis started with exercises in the skills lab using organs of the pig (Fig. 3). First, end-to-end anastomosis of the small bowel and the colon was trained. The next exercise was cross-section gastroenterostomy (Fig. 3B) and gastroenterostomy with Braun anastomosis. Lastly, the anastomosis of the descending colon and upper rectum was trained with a circular stapler. In a next step, parts of the skills lab exercises were transferred to the animal surgery. The in vivo exercises consisted of end-to end anastomosis of the small bowel (Fig. 3C), cross-section gastroenterostomy (Fig. 3D) and anastomosis of the descending colon and upper rectum.

### Techniques in vascular surgery

**Fig. 4:**
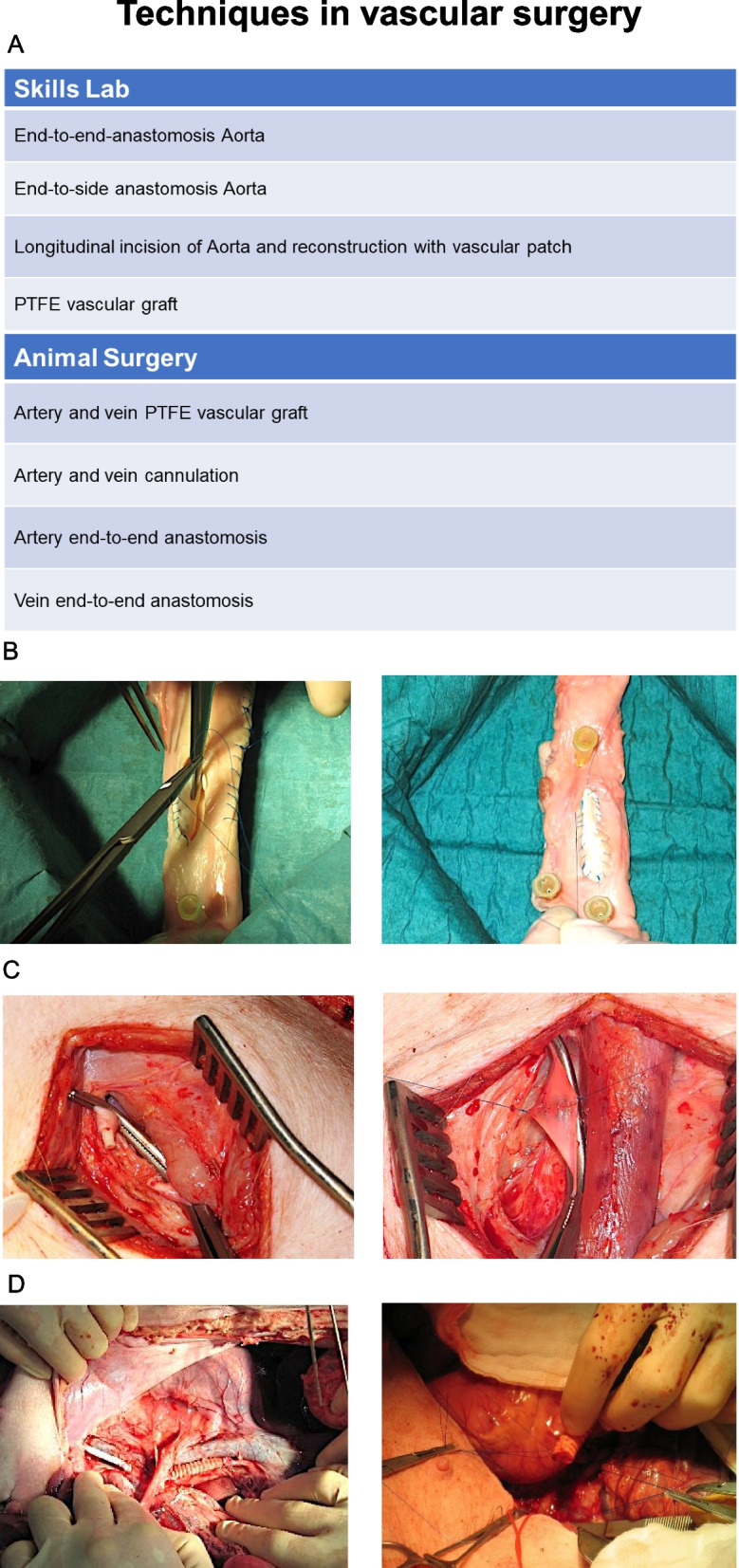
Techniques in vascular surgery. **A** Table of exercises in the skills lab ex vivo and animal surgery in vivo. **B** Exemplary picture of the exercise “Longitudinal incision of the Aorta with vascular patch” **C** Exemplary picture of the exercise “end-to-end anastomosis” (in vivo) **D** Exemplary picture of the exercise “PTFE vascular graft” (in vivo)

The module techniques in vascular surgery started in the skills lab with the exercises end-to-end- and end-to-side anastomosis of the Aorta (Fig. 4). Next, the longitudinal incision of the Aorta and the reconstruction with vascular patches was trained, followed by replacement of vessels with PTFE vascular grafts (Fig. 4B). During animal surgery training, participants could perform end-to-end anastomosis (Fig. 4C) and cannulation of the artery and vein artery as well as artery and vein reconstruction with PTFE vascular graft (Fig. 4D).

#### Animals and organs for training

All experiments involving living animals were performed in accordance with the German Animal Welfare Law and beforehand approved by the administrative authority of North Rhine-Westphalia (reference numbers 9.93.2.10.55.07.048, 8.84–02.05.30.11.044 and 84–02.05.40.14.083). Female pigs weighing 20 – 60 kg were obtained from a local farmer and transferred to the central animal facility of the University Muenster for an acclimatization period of at least 7 days.

We used a previously described standardized anesthesia protocol [16]. After fasting for 12 h (with free access to water), the animals were premedicated with intramuscular injections of azaperon (2 mg/kg body weight; Elanco, Bad Homburg, Germany), ketamine (15 mg/kg body weight; WDT, Garbsen, Germany), and atropine (10 µg/kg body weight; B. Braun Melsungen AG, Melsungen, Germany). After the first venous access was established by cannulation of the ear vein, propofol (3 mg/kg body weight; Fresenius Kabi Deutschland GmbH, Bad Homburg, Germany) was used to initiate anesthesia, followed by endotracheal intubation. Anesthesia was maintained using isoflurane (1.5 vol%; Baxter, Deerfield, IL, USA). Analgesia was ensured by continuous perfusion with fentanyl (0.005 mg/kg body weight, Rotexmedica, Trittau, Germany). The animals were monitored using a pulse oximeter and body temperature probe (rectal probe).

Organs for ex vivo training were ordered by a local slaughterhouse and freshly picked on the day of the course.

#### Statistical analysis

Data were collected using Microsoft Excel 2010 (Microsoft Corporation, Redmond, WA, USA) and analyzed using GraphPad Prism 10 (GraphPad Software, San Diego, CA, USA). Descriptive statistics were used to depict the number of participants and results of the self-assessment. The results of the self-assessment five-point scale are described as mean and standard deviation (SD). Statistical analysis was performed to compare the pre- and post-course results for each exercise using a t-test for paired samples. Statistical significance was set at *p* < 0.05.

## Results

**Fig. 5:**
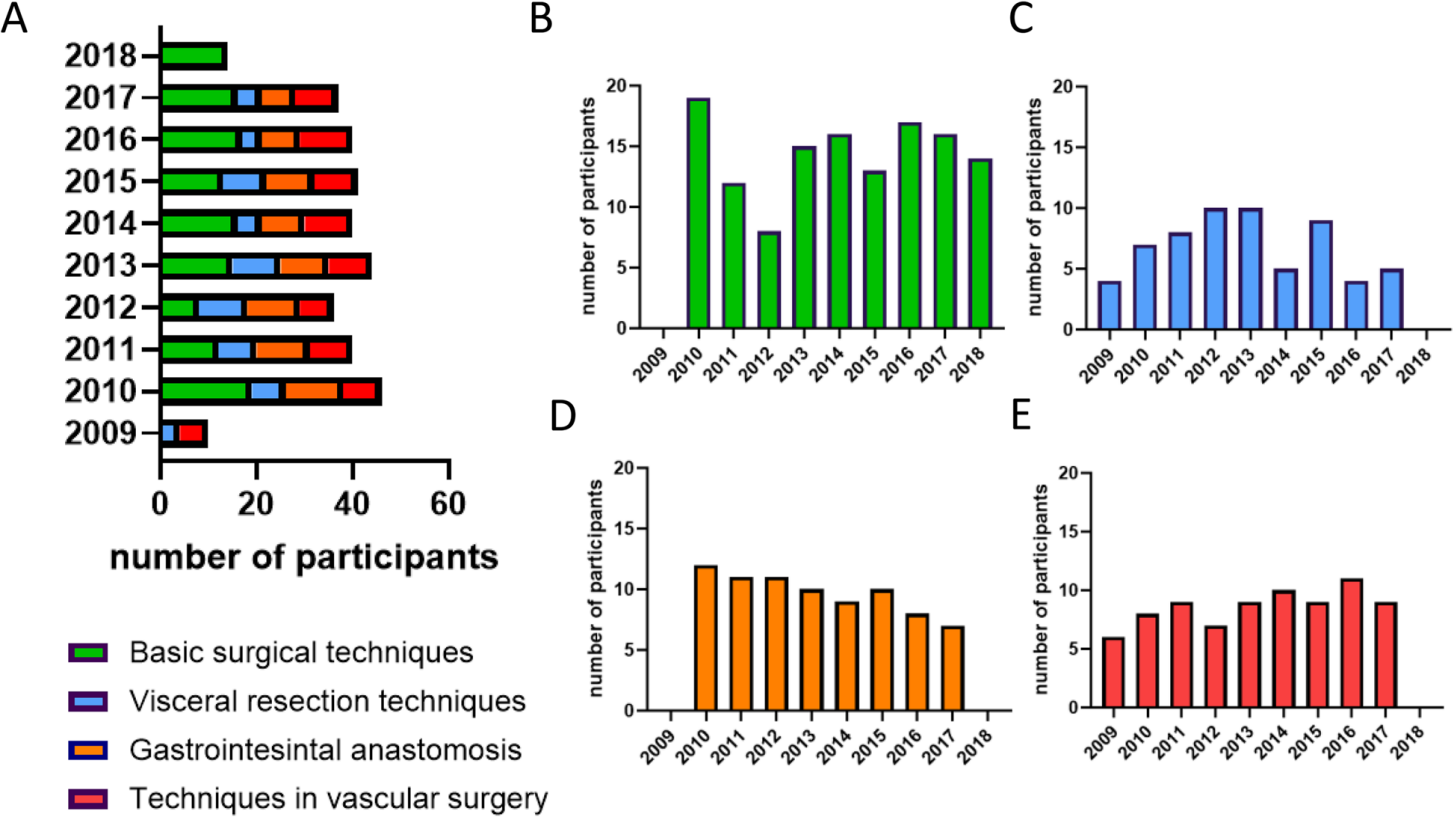
Participants of the Basic Surgical Skills training. **A** Overview of the number of participants from 2009 to 2018 in the different modules “ Basic surgical techniques” (**B**), “ Visceral resection techniques” (**C**), “ Gastrointestinal anastomosis” (**D**) and “ Techniques in vascular surgery”

Over ten years, 348 individuals successfully completed the Basic Surgical Skills training program. Of these, 130 (37.4%) participants completed the module basic surgical techniques, 62 (17.8%) visceral resection techniques, 78 (22.4%) gastrointestinal anastomosis, and 78 (22.4%) completed the module of techniques in vascular surgery (Fig. 5).

All participants completed a self-evaluation questionnaire before and after the course, and we consecutively evaluated whether the completion of the course resulted in an improvement in their surgical skills based on their self-assessment.

### Basic surgical techniques

**Fig. 6:**
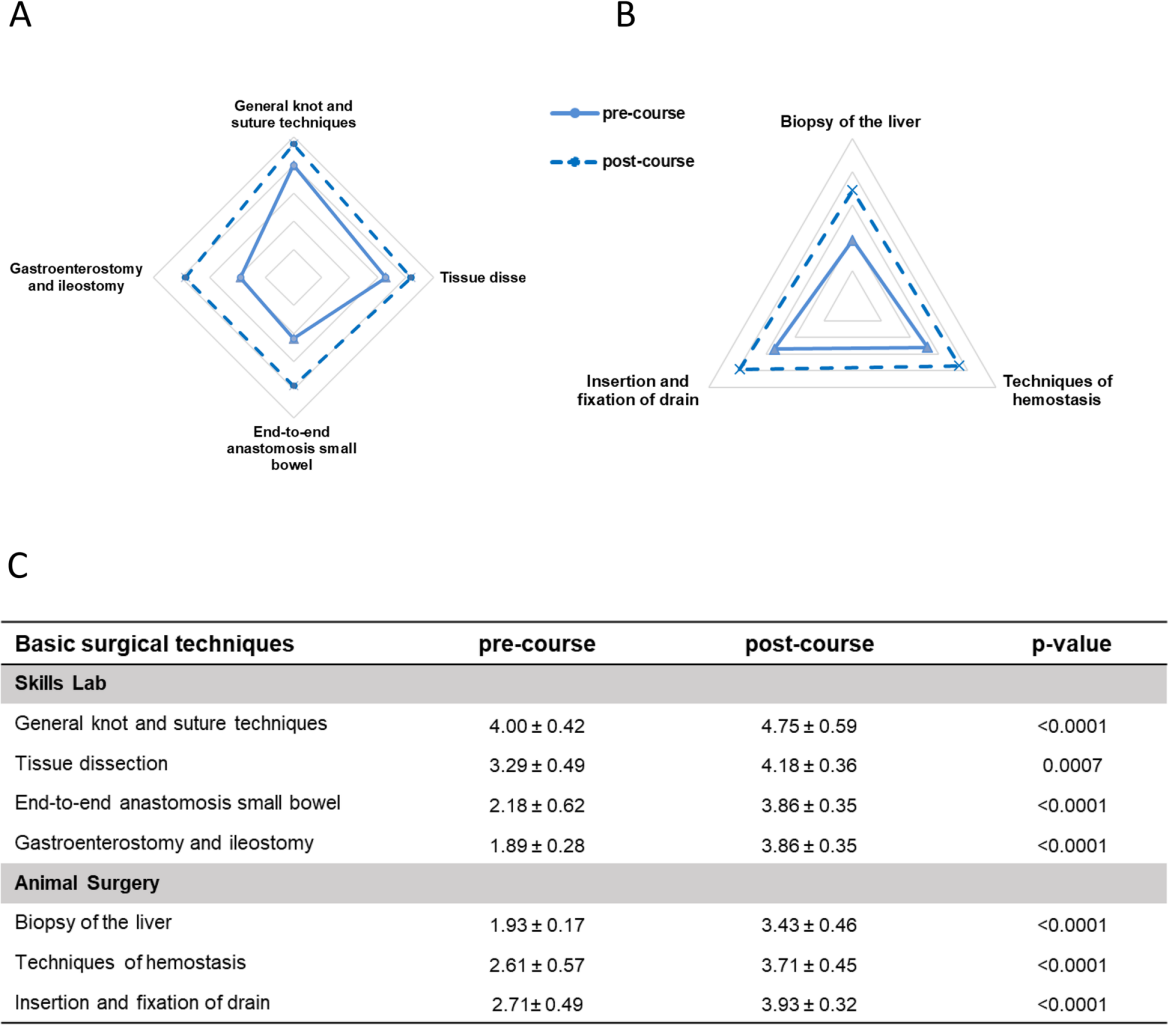
Pre- and post-course self-assessments of participants attending module *Basic surgical techniques.* Spider web graphs display the differences between the pre- (full line) and post-course (dotted line) self-assessments. **A** Exercises in the skills lab **B** Exercises during animal surgery. **C** Table of pre- and post-course self-assessments with a five-step scale, with 1 = no theoretical or practical knowledge, 2 = no practical but theoretical knowledge, 3 = performance with major help, 4 = independent performance with little help, and 5 = independent performance without help. Data are presented as mean ± standard deviation (SD) and compared using Student’s t-test for paired samples. A *p*-value < 0.05 was considered statistically significant

In the four exercises trained in the skills lab, we could find significant higher scores after the course when comparing pre- and post-course self-assessment (Fig. 6A, C, Supplementary Fig. 2). Similarly, the majority of the participants gained experience and knowledge on how to perform biopsies of the liver, techniques of hemostasis and insertion and fixation of a drain during animal surgery resulting in significant higher scores after the course (Fig. 6B, C, Supplementary Fig. 2).

### Visceral resection techniques

**Fig. 7:**
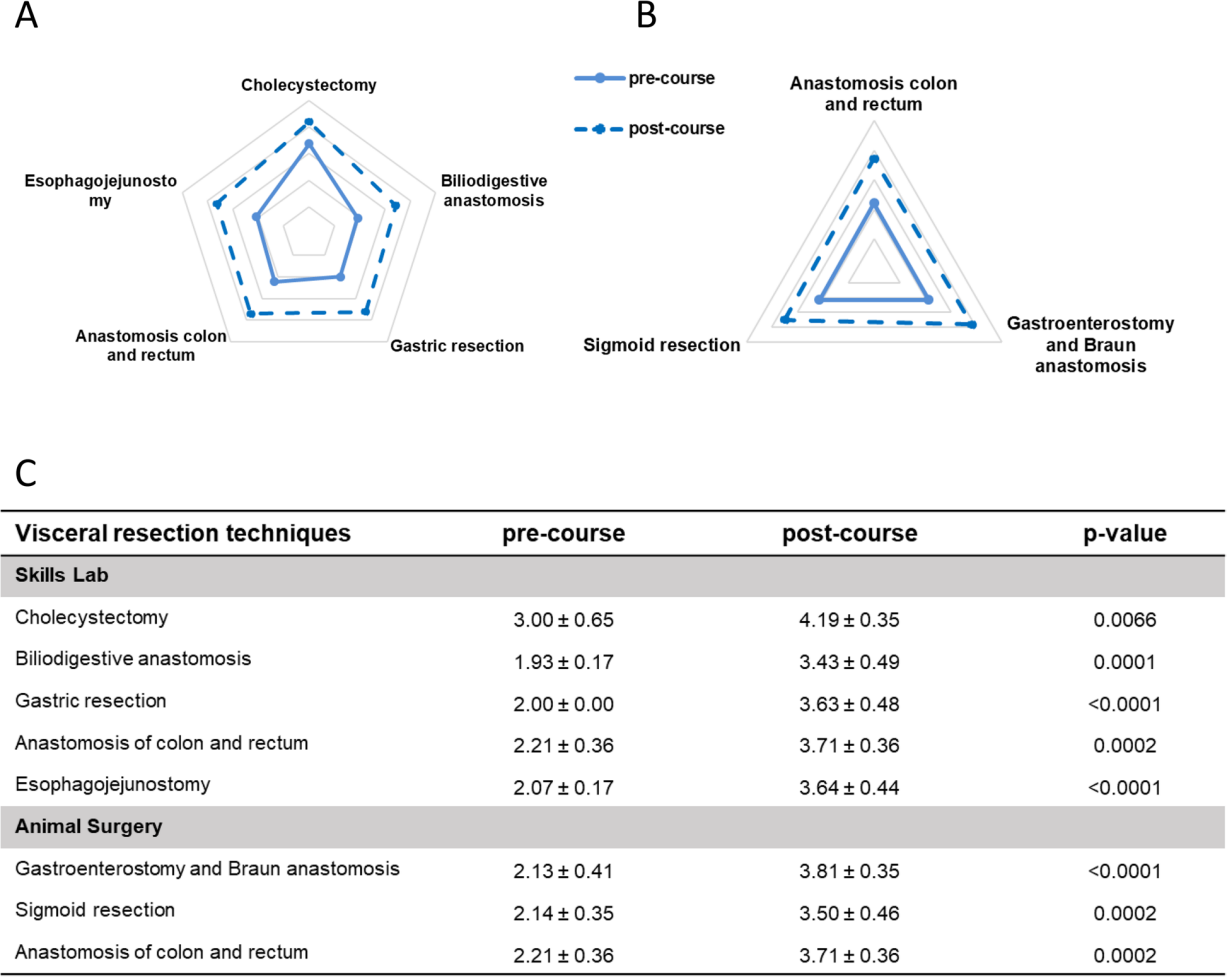
Pre- and post-course self-assessments of participants attending the module *Visceral resection techniques*. Spider web graphs display the differences between the pre- (full line) and post-course (dotted line) self-assessments. **A** Exercises in the skills lab **B** Exercises during animal surgery. **C** Table of pre- and post-course self-assessments with a five-step scale, with 1 = no theoretical or practical knowledge, 2 = no practical but theoretical knowledge, 3 = performance with major help, 4 = independent performance with little help, and 5 = independent performance without help. Data are presented as mean ± standard deviation (SD) and compared using Student’s t-test for paired samples. A *p*-value < 0.05 was considered statistically significant

The analysis of self-assessment for the surgical training of visceral resection techniques revealed that the majority of the participants gave lower scores before the training (Fig. 7, Supplementary Fig. 2). In detail, we could see a significant higher score for the five evaluated skill lab exercises after the course (Fig. 7A, C). Similar results were found for the exercises gastroenterostomy with Braun anastomosis, sigmoid resection and anastomosis of colon and rectum trained in animals with significant higher scores after the training (Fig. 7B, C).

### Gastrointestinal anastomosis

**Fig. 8:**
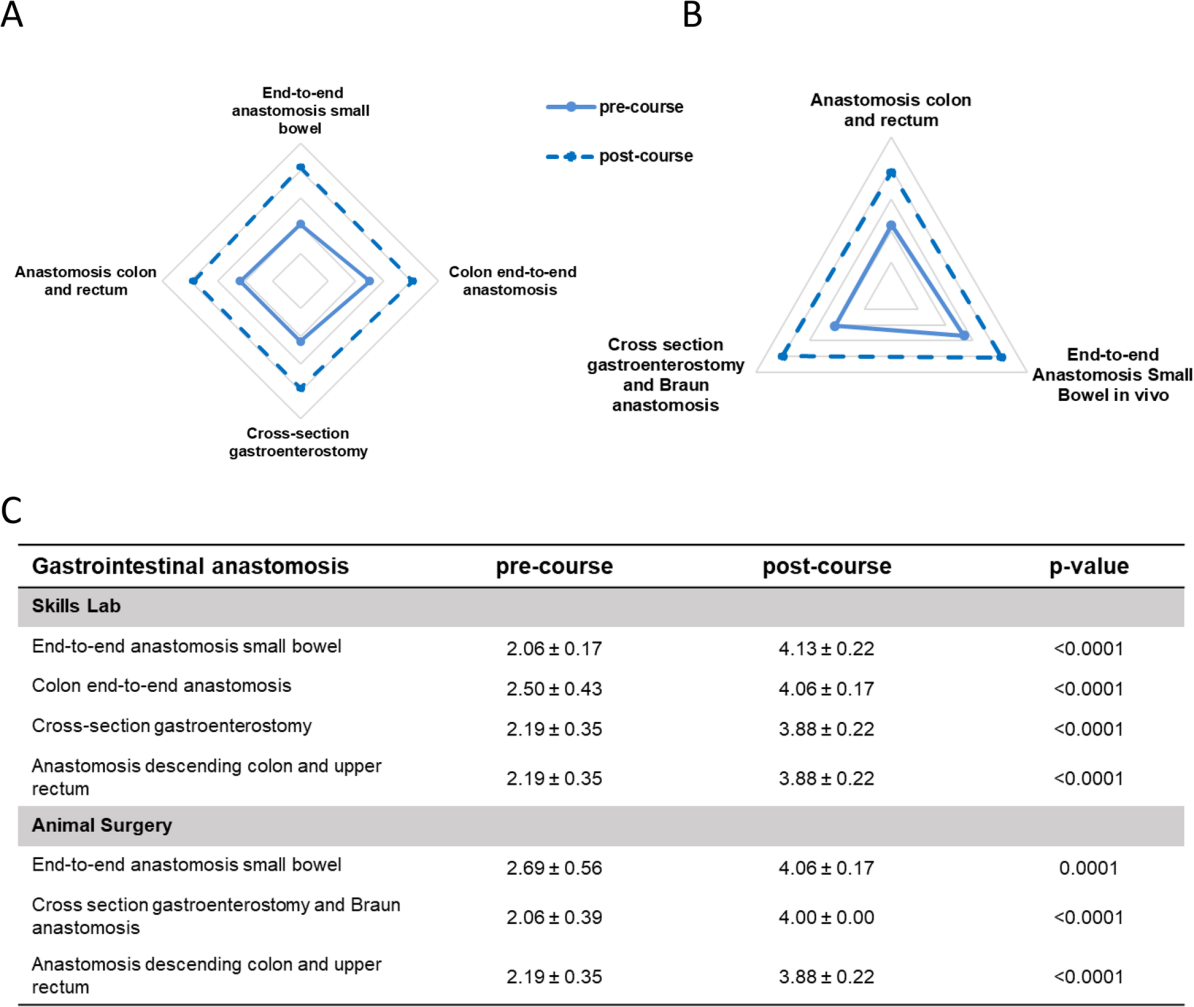
Pre- and post-course self-assessments of participants attending the module *Gastrointestinal anastomosis*. Spider web graphs display the differences between the pre- (full line) and post-course (dotted line) self-assessments. **A** Exercises in the skills lab **B** Exercises during animal surgery. **C** Table of pre- and post-course self-assessments with a five-step scale, with 1 = no theoretical or practical knowledge, 2 = no practical but theoretical knowledge, 3 = performance with major help, 4 = independent performance with little help, and 5 = independent performance without help. Data are presented as mean ± standard deviation (SD) and compared using Student’s t-test for paired samples. A *p*-value < 0.05 was considered statistically significant

Upon analyzing the surgical training for gastrointestinal anastomosis, we observed that the participants generally assessed their skills before the course regarding the exercises both in skills lab and animal surgery with low scores (score: 2.06 – 2.69, Fig. 8C, Supplementary Fig. 2). However, after the skills lab training, we could see a significant increase in score values (Fig. 8A, C). Comparable results were seen in the exercises of animal surgery with significant higher scores after the training (Fig. 8B, C).

### Techniques in vascular surgery

**Fig. 9:**
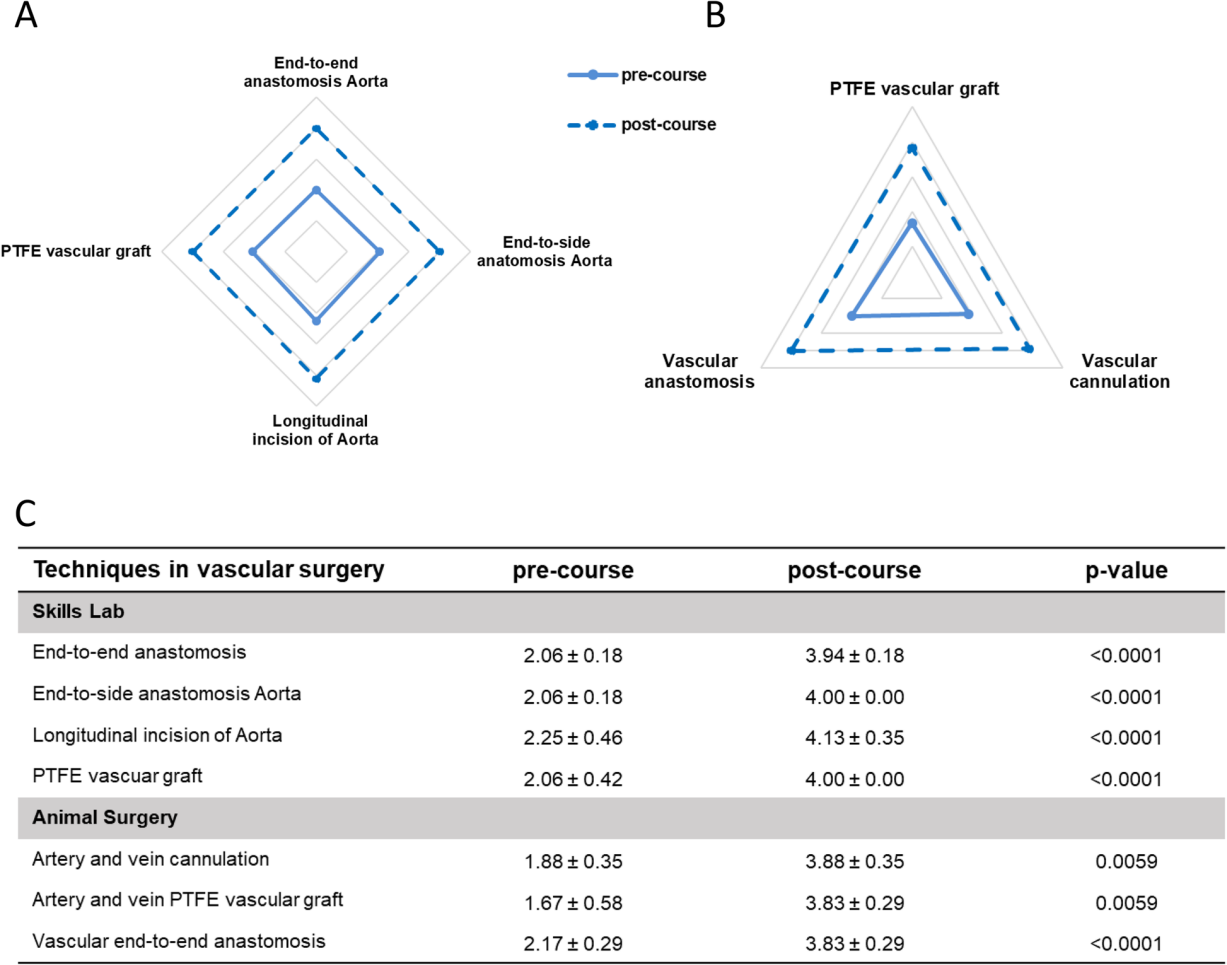
Pre- and post-course self-assessments of participants attending module *Techniques in vascular surgery*. Spider web graphs display the differences between the pre- (full line) and post-course (dotted line) self-assessments. **A** Exercises in the skills lab **B** Exercises during animal surgery. **C** Table of pre- and post-course self-assessments with a five-step scale, with 1 = no theoretical or practical knowledge, 2 = no practical but theoretical knowledge, 3 = performance with major help, 4 = independent performance with little help, and 5 = independent performance without help. Data are presented as mean ± standard deviation (SD) and compared using Student’s t-test for paired samples. A *p*-value < 0.05 was considered statistically significant

For the module techniques in vascular surgery, the self-assessment analysis revealed that the participants assessed their skills with a low scoring (score: 1.67 – 2.25, Fig. 9C, Supplementary Fig. 2) before the course. However, after completion of the skills lab exercises, the participants stated that they performed the majority of the exercises independently with minor support (scores of 3.94—4.13) with significant higher scores post-course (Fig. 9A, C). In addition, a comparison of surgical skills before and after the course for the exercises with animals showed a significant higher score in all exercises after the course (Fig. 9B, C).

## Discussion

Properly structured and adequately funded surgical education is highly beneficial for both trainees and hospitals, as well as university departments, as it enables high-quality surgical procedures while ensuring patient safety. Here, we present our 10-year experience with the Basic Surgical Skills curriculum developed and implemented by the Department of General, Visceral and Transplant Surgery, University Hospital Muenster. In our course, we pursue three main educational principles: 1. Theory before praxis – a fundamental comprehension of the theoretical background is essential for the successful implementation of praxis; 2. Step-by-step – It is important to have a full understanding of each step to cumulatively perform a complicated surgical procedure; 3. Hands-on training begins with synthetic materials or isolated animal organs, progressing from inorganic to organic substances.

In the present study, all participants evaluated their surgical skills in the different modules of the course using a self-assessment questionnaire, and we clearly demonstrated a gain in their practical skills. Independent of the foreknowledge of the participants, each of them successfully performed different surgical tasks under the guidance of experienced surgeons to acquire additional knowledge and to train their surgical skills regarding the different steps performed with the result of significant improvement.

In general, simulation can shorten the learning curve and provide trainees with the required skills and confidence necessary to perform complex surgical procedures [10, 11]. Furthermore, simulations can reduce costs and improve safety and quality [10]. Currently, surgical education in many countries is increasingly focusing on simulation-based training [17–22]. For centuries, surgical education has been the traditional model of learning through observation and assistance. The trainee worked in a hospital setting, and surgical judgment and technical skills were learned on the job by following the mentor (senior surgeon) [23]. This traditional model has been outdated in the modern era of evidence-based and outcome-based medicine owing to the emphasis on patient safety and surgeon performance [24–26]. Thus, in surgical education, simulation-based training tools such as artificial models have endorsed traditional teaching to complement training modalities, allowing for surgical skill enhancement while reducing pressure on trainees and trainers [26, 27].

Our results show that the participants gained knowledge of surgical procedures, indicating an improvement in surgical skills in our multi-step surgical curriculum combining ex vivo with in vivo training. In fact, the participants were able to perform specific exercises autonomously at the end of the course. This finding highlights the substantive impact of simulation training and the associated increase in self-confidence through mentor-guided teaching. Similar results have been shown in other studies demonstrating positive effects especially in acquiring technical skills and surgical competence [6, 28–33]. Several tools are available for surgeons to simulate an operation, including bench models, cadavers, animal models, and augmented and virtual reality [23, 34, 35]. However, most training methods focus on a single method or approach disregarding the complexity of surgical competence development [6]. As surgical procedures and associated skills become more complex, the challenge of implementing appropriate simulation training has increased [36]. Additionally, the transfer of acquired skills to the operating room occasionally remains challenging [37–39]. In the present study, bench models and animal organs were used to teach simple, single steps of complicated surgery. The use of live animals serves as the last step in applying the already acquired knowledge to an in vivo setup. Therefore, the participants were able to learn about tissue handling and hemostasis. The setting resembled the operating room; thus, participants learned how to work together in teams, help one another, and communicate effectively, thus, focusing on non-technical skills important for surgical competence development [6]. Due to the need for a comprehensive infrastructure, several novel regulations and significant pressure from animal protection organization and the government, the implementation of animal vivisection especially in large animals is nowadays restricted to few facilities in the country.

There is no doubt that this study has some limitations. Surgical organizations require costly equipment such as surgical instruments and devices, as well as a fully equipped operating room for the in vivo setup. Cooperation with outside companies may be needed to solve equipment issues and reduce costs. An additional limitation of the course was its brief duration, as it only spanned a single day. The participants may not have had sufficient time to repeat the different surgical procedures. Regarding the questionnaire for the self-assessment of the participants, it must be mentioned that it has not been validated, thus, limiting the validity of the results obtained herein. Assessment using standard and objective metric measurements strengthens the quality of surgical education, in particularly when providing feed-back to trainees [40, 41]. Thus, several surgical skill measurement techniques exist including questionnaires and post-training surveys, objective structured assessments of technical skills, global rating scales, motion tracking and video recording analysis [40]. As surgical skill training is increasingly incorporated into surgical education, there is an urgent need to structure and standardize techniques to adequately measure skill proficiency [40, 42].

Our assessment conducted as part of the course unequivocally highlighted the ability of our program to impart short-term skills to participants. However, it would have been beneficial to administer a post-course survey to gauge the impact of the program on the professional trajectories of its graduates. It can only be assumed that the course enabled the participants to conduct complex surgical procedures at their home institutions.

We can conclude that our curriculum, which was structured in a step-by-step manner, enabled us to develop a highly effective training program for junior and assistant doctors to learn challenging surgical techniques through ex vivo exercises, and subsequently apply this knowledge and manual dexterity to live surgical procedures.

## Supplementary Information

Supplementary Material 1: Fig. 1 Questionnaire Basic surgical techniques. Fig. 2 Questionnaire Visceral resection techniques. Fig. 3 Questionnaire Gastrointestinal anastomosis. Fig. 4 Questionnaire Techniques in vascular surgery. Fig. 5 Pre- and post-course self-assessments of participants showing mean score with a five-step scale, with 1 = no theoretical or practical knowledge, 2 = no practical but theoretical knowledge, 3 = performance with major help, 4 = independent performance with little help, and 5 = independent performance without help and average improvement of participants for each exercise A. Basic surgical techniques B. Visceral resection techniques C. Gastrointestinal anastomosis D. Techniques in vascular surgery. Significant differences have not been depicted for clarity and can be found in the corresponding tables (Figs.6, 7, 8 and 9).

## Data Availability

The datasets used and/or analyzed during the current study are available from the corresponding author on reasonable request.

## Abbreviation

PTFE: Polytetrafluoroethylene
