# Supplementary material for: Development and evaluation of a surgical skills lab for trainee surgeons: a 10-year experience at the Münster University Hospital
Source: BMC Med Educ. 2025 Apr 4;25:484. doi: 10.1186/s12909-025-07064-3 (PMC11971789; doi:10.1186/s12909-025-07064-3)
Supplement: Supplementary file 1 — Supplementary Material 1: Fig. 1 Questionnaire Basic surgical techniques. Fig. 2 Questionnaire Visceral resection techniques. Fig. 3 Questionnaire Gastrointestinal anastomosis. Fig. 4 Questionnaire Techniques in vascular surgery. Fig. 5 Pre- and post-course self-assessments of participants showing mean score with a five-step scale, with 1 = no theoretical or practical knowledge, 2 = no practical but theoretical knowledge, 3 = performance with major help, 4 = independent performance with little help, and 5 = independent performance without help and average improvement of participants for each exercise A. Basic surgical techniques B. Visceral resection techniques C. Gastrointestinal anastomosis D. Techniques in vascular surgery. Significant differences have not been depicted for clarity and can be found in the corresponding tables (Figs.6, 7, 8 and 9). [file 12909_2025_7064_MOESM1_ESM.pdf]

Self –assessment of performed skills

1 = no theoretical or practical knowledge
2 = no practical but theoretical knowledge
3 = performance with major help
4 = independent performance with little help
5 = independent performance without help

Basic surgical techniques	before					after				
Skills Lab	1	2	3	4	5	1	2	3	4	5
General knot and suture techniques										
Tissue dissection										
End-to-end anastomosis small bowel										
Gastroenterostomy and ileostomy										
Animal Surgery	1	2	3	4	5	1	2	3	4	5
Biopsy of the liver										
Techniques of hemostasis										
Insertion and fixation of drain										

Supplementary Figure 1

Self –assessment of performed skills

1 = no theoretical or practical knowledge
2 = no practical but theoretical knowledge
3 = performance with major help
4 = independent performance with little help
5 = independent performance without help

Visceral resection techniques	before					after				
Skills Lab	1	2	3	4	5	1	2	3	4	5
Chlocystectomy										
Biliodigestive anastomosis										
Gastric resection										
Anastomosis of colon and rectum										
Esophagojejunostomy										
Animal Surgery	1	2	3	4	5	1	2	3	4	5
Gastroenterostomy and Braun anastomosis										
Sigmoid colon resection										
Anastomosis of colon and rectum										

Supplementary Figure 2

Self –assessment of performed skills

1 = no theoretical or practical knowledge
2 = no practical but theoretical knowledge
3 = performance with major help
4 = independent performance with little help
5 = independent performance without help

Gastrointestinal anastomosis	before					after				
Skills Lab	1	2	3	4	5	1	2	3	4	5
End-to-end anastomosis small bowel										
Colon end-to-end anastomosis										
Cross-section gastroenterostomy										
Anastomosis colon and rectum										
Animal Surgery	1	2	3	4	5	1	2	3	4	5
End-to-end anastomosis small bowel										
Gastroenterostomy and Braun anastomosis										
Anastomosis colon and rectum										

Supplementary Figure 3

Self –assessment of performed skills
1 = no theoretical or practical knowledge
2 = no practical but theoretical knowledge
3 = performance with major help
4 = independent performance with little help
5 = independent performance without help

Techniques in vascular surgery	before					after				
Skills Lab	1	2	3	4	5	1	2	3	4	5
End-to-end anastomosis Aorta										
End-to-side anastomosis Aorta										
Longitudinal incision Aorta										
PTFE vascular graft										
Animal Surgery	1	2	3	4	5	1	2	3	4	5
Artery and vein cannulation										
Artery and vein PTFE vascular graft										
Vascular end-to-end-anastomosis										

Supplementary Figure 4

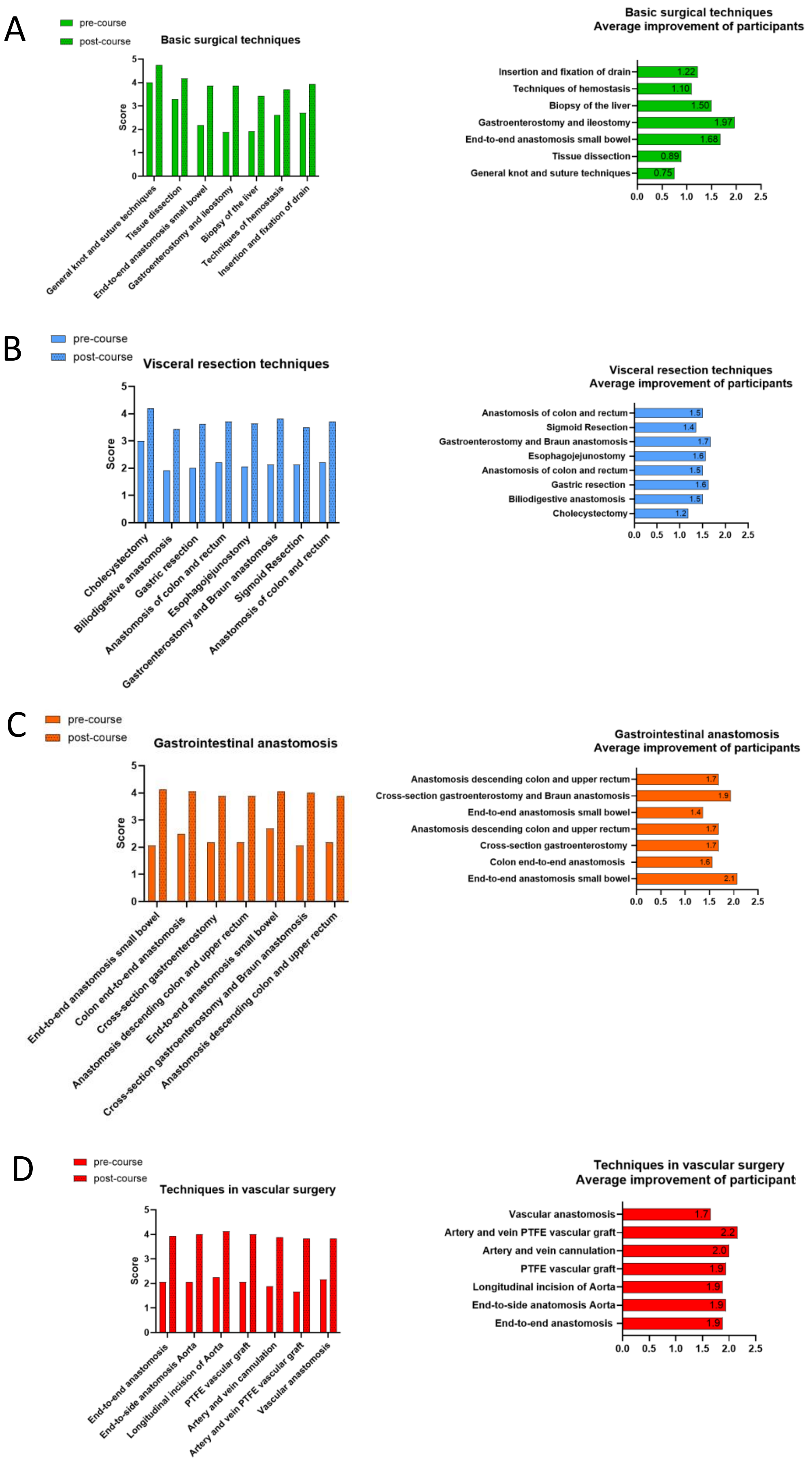

Supplementary Figure 5
